# Ereptiospiration

**DOI:** 10.3390/bioengineering4020033

**Published:** 2017-04-12

**Authors:** Christine Woolley, Antonio A. Garcia, Marco Santello

**Affiliations:** School of Biological and Health Systems Engineering, Arizona State University, Tempe, AZ 58287-6006, USA; Christine.grewe@asu.edu (C.W.); marco.santello@asu.edu (M.S.)

**Keywords:** thermal transpiration, Knudsen, Knudsen number, acetaminophen, coconut oil, lanolin, vaporization, rapid vaporization, thermal lability, Kanthal, thermal degradation

## Abstract

Pure coconut oil, lanolin, and acetaminophen were vaporized at rates of 1–50 mg/min, using a porous network exhibiting a temperature gradient from 5000 to 5500 K/mm, without incurring noticeable chemical changes due to combustion, oxidation, or other thermally-induced chemical structural changes. The newly coined term “ereptiospiration” is used here to describe this combination of thermal transpiration at high temperature gradients since the process can force the creation of thermal aerosols by rapid heating in a localized zone. Experimental data were generated for these materials using two different supports for metering the materials to the battery powered coil: namely, a stainless steel fiber bundle and a 3-D printed steel cartridge. Heating coconut oil, lanolin, or acetaminophen in a beaker to lower temperatures than those achieved at the surface of the coil showed noticeable and rapid degradation in the samples, while visual and olfactory observations for ereptiospiration showed no noticeable degradation in lanolin and coconut oil while HPLC chromatograms along with visual observation confirm that within the limit of detection, acetaminophen remains chemically unaltered by ereptiospiration.

## 1. Introduction

Recent advances in scanning calorimetry have expanded the ability to take thermal property measurements of compounds that decompose at temperatures lower than the threshold values for melting or vaporization. Ultra-fast differential scanning and fast scanning chip calorimeters have demonstrated very rapid heating rates [1] on the order of several thousand Kelvin per second, and the chip-based platform can conduct an entire measurement for a particular sample within a fraction of a second, thus yielding information on the reversible conversion of beta-pleated crystal structures of silk fibroin to random coils and helices [2] as well as the determination of ionic liquid vaporization enthalpies [3]. Given these practical demonstrations of the principle that decomposition has finite kinetics, if temperature-induced physical changes occur rapidly enough, some compounds that are currently considered to be too labile for vaporization may in fact be amenable to vaporization if a suitable, rapid means of heating is used.

There are many compounds in pharmacology that are characterized as undergoing decomposition at temperatures lower than their boiling point [4]. As a result, therapeutic applications rely on drug administration as a solid, a suspension, or as an injectable formulation, all requiring appropriate mixing with additional compounds, modifiers, and/or solvents. Pulmonary inhalation requires forming a powder or mixing in an appropriate solvent that can be aerosolized using a nebulizer or fine mist spray system. However, some medications may be more desirably delivered to the lungs in a pure form in order to lower the therapeutic dose, speed the therapeutic action, and/or minimize unwanted effects of potentially harmful additives [5]. Previous thinking that vaporization of pure labile compounds would not be possible did not consider the possibilities of rapid heating suggested by the above mentioned chip-based calorimeters, thermal aerosols made by rapid heating (0.5 s) of a thin film [6], or by the new fast vaporization method described here as “ereptiospiration” which is a combination of the latin words ereptio for “forcible taking” and spirare for “to breathe”.

In ereptiospiration, a compound can be a solid or viscous liquid that is heated to flow and subsequently rapidly vaporizes using very large temperature gradients within a specially constructed porous material or microchannel device. By analogy with fast scanning calorimetry (e.g., flowing the compound rapidly through microchannels across a temperature gradient of several thousand Kelvin per millimeter) exposure to high temperatures is limited to very short times. To ensure that the labile compound undergoes a controlled flow so that the high temperature exposure is maintained at millisecond or faster time scales, it is desirable to have the high temperature zone-controlled metering not based on continuum fluid mechanics, but rather in the transitional or Knudsen flow regimes where the temperature difference across the channel creates a pressure-driving force in the vapor phase. This phenomenon has been used in studies of thermal transpiration [7,8,9,10,11,12] and in heat exchange devices with no moving parts [13]. However, unlike thermal transpiration devices, ereptiospiration does not rely on molecular flow for functionality, but instead can be operated at lower Knudsen numbers due to the use of high temperature gradients.

This paper reports on our exploration of three model compounds: lanolin, coconut oil, and acetaminophen [14]. Experiments show that these compounds can be forced into the vapor phase without apparent decomposition using porous stainless steel structures either fabricated via 3-D printing or constructed from extra fine stainless steel wool. We also report that the power needed for vaporization at rates on the order of milligrams per minute can be supplied by a rechargeable battery, and that accordingly, the heating element is a relatively thin or high gauge wire. Visual and olfactory observations of lanolin and coconut oil ereptiospiration suggest that they form aerosol droplets that are undecomposed. High pressure liquid chromatography analysis of acetaminophen before and after ereptiospiration indicate that there are no detectable changes in its chemical composition due to this vaporization process.

## 2. Materials and Methods

### 2.1. Design and Fabrication of a Fiber Bundle System and Substances Used to Demonstrate Ereptiospiration

Fiber bundles that transport the material to be rapidly vaporized were made using stainless steel. Briefly, a cluster of fine grade stainless steel fibers (International Steel Wool, Roseville, MI, USA) was rolled tightly to form fiber bundles having a diameter of between 0.8 and 1 mm and cut to a length of 75 mm. The weight of the fiber bundles used in the experiments averaged 0.7 ± 0.04 grams. A variety of liquids (Table 1) was used to characterize the action of the fiber bundles. The ability of the processed fiber bundle to take up liquid through capillary action when the fiber bundle is dry is summarized in Table 2. In order to accomplish the fast vaporization needed in ereptiospiration, we used a 34-gauge kanthal wire to form a coil at the center of the fiber bundle using 12 turns around the fiber bundle.

### 2.2. Design and Fabrication of Cartridge

**Figure 1:**
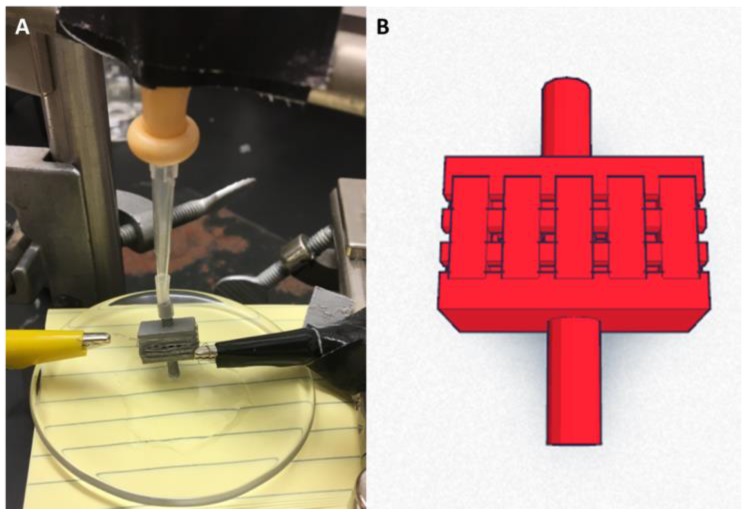
(**A**) Image of the 3D printed cartridge for ereptiospiration testing; and (**B**) screen shot of the 3D drawing used to create the cartridge.

A photograph of the cartridge used (side view), as well as a schematic diagram, are shown in Figure 1. The stainless steel device was designed using Fusion 360 (Autodesk Inc., San Rafael, CA, USA) and a 3-D printer (iMaterialise, Leuven, Belgium). Inlet channels were designed to have a length of 8 mm extending below the cartridge body and an opening with a 1-mm diameter. The body of the cartridge contains a series of networked pores of 0.5-mm and 1-mm radii through which heated material is brought to the surface of the coil where ereptiospiration takes place.

This cartridge was used for all experiments. In each experimental setup, the heating was accomplished by tightly wrapping a 34-gauge kanthal wire of 155 mm length around the exterior body of the cartridge. The coil was held in place using alligator clips which were connected to a rechargeable 3.6-V battery (Li-ion power cell, can be charged to a maximum of 4.1 volts) used to heat the coil and provide power to the circuit. The second inlet channel was plugged using a 55-mm piece of tubing stopped with a pipet tip and bulb (Figure 1A). This was done to prevent air to flow in the other tube once power was supplied, allowing the substance being tested to rise into the cartridge from the inlet and be exposed to the high temperatures created at the coil/cartridge interface.

### 2.3. Data Collection

Data was collected to monitor ereptiospiration of substances using both the stainless steel fiber bundle/coil assembly, as well as the stainless steel cartridge. In both of these configurations, the material being studied undergoes a rapid phase change where material is forced into the air very rapidly so that the time spent in the high temperature zone is minimized and decomposition does not take place [2].

#### 2.3.1. Fiber bundle and Coil

**Figure 2:**
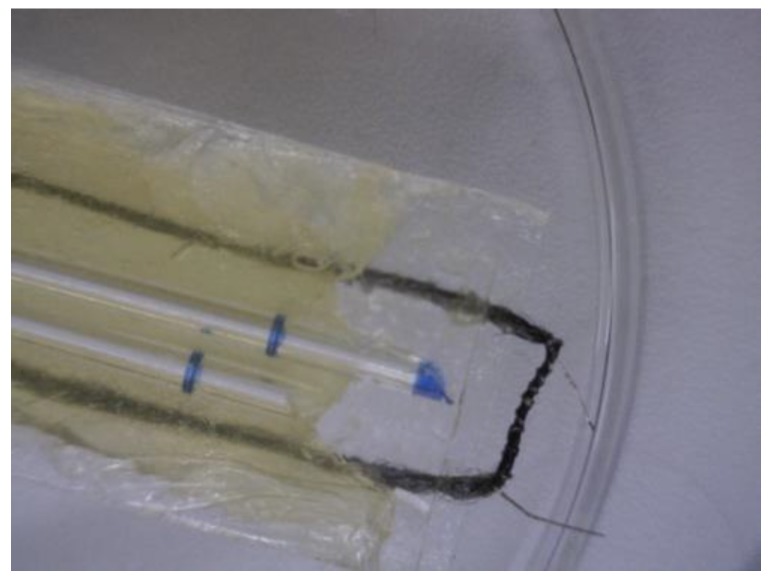
Fiber bundle and coil system shown loaded with lanolin. The fiber bundle is sandwiched between two glass slides and the capillary tubes are used to create a space between the slides in order to maintain a reservoir of lanolin. The coil and a section of the fiber bundle is maintained free from direct contact with the lanolin in order to minimize flow of material directly to the coil from outside of the fiber bundle pores.

The coil was wrapped tightly around the fiber bundle and the fiber bundle ends were placed into the substance being tested (Figure 2). As power was supplied to the coil, temperatures measured using a K-type thermocouple gave a reading of up to 185 °C. However, since the thermal mass of the coil is much lower than the thermocouple, the maximum temperature at the coil surface is much higher and cannot be measured. Appendix A provided with this paper explains in more detail the surface temperature calculations based on power supplied. The bulk rate of rapid ejection into the vapor phase was determined by weight difference over time and capture of condensed material with lanolin, coconut oil, and acetaminophen samples.

#### 2.3.2. Cartridge

A 34-gauge Kanthal coil was wrapped tightly around the cartridge and secured in place as described above (Figure 1A). One of the cartridge’s two ports was placed into the substance to be tested (using a watch glass as an inlet reservoir) which allowed liquid material to fill the tube via capillary action (see Table 2). Once power was supplied to the coil wrapped around the cartridge, the surface of the cartridge that contained the coil was found to reach temperatures of 180–200 °C, using a K-type thermocouple. Again, as noted above, the actual coil surface temperature could not be measured due to its low thermal mass compared to that of the thermocouple. Visual and auditory observations confirmed that after approximately 10 s, there was removal of the material from the watch glass at a section of the coil near a pore entrance due to suction of the material from the port to the surface of the cartridge. This pressure difference is required for ereptiospiration since the material needs to be drawn to the cartridge surface from the port, a distance of between 2 and 4 mm.

#### 2.3.3. High Pressure Liquid Chromatography

The high pressure liquid chromatograph used for analyzing acetaminophen was an Agilent 1100 Series HPLC value system (Santa Clara, CA, USA) and consisted of an Agilent Binary pump G1312A (serial # DE40915180), a Rheodyne Model 7725i injector equipped with a 20-µL loop, and an Agilent Variable Wavelength Detector (VWD) G1314A (serial # DE51530425, wavelength at 254 nm). System control and results display were accomplished using an Agilent ChemStation for LC system software. The column used was a commercially available stainless steel HPLC column with a length of 15 cm and inside diameter of 4.6 mm packed with Keystone BDS C18 (Sigma-Aldrich, St. Louis, MO, USA) stationary phase. The mobile phase was a mixture of water–methanol–glacial acetic acid (690:280:30) and was found to maintain an apparent pH of 4.7 for several weeks. The mobile phase mixture was degassed using a helium sparger. For all chromatograms, the mobile phase flow rate was 1.0 mL/min, isocratic elution was employed, and the column temperature was the same as ambient temperature.

## 3. Results

Both porous systems with heating coils were evaluated for the amount of material that could be drawn in through capillary action while at room temperature and for coconut oil at 50 °C (Table 2). This was done to determine if capillary action alone was responsible for delivering the material to the coil. For both the fiber bundle/coil and coil/cartridge systems, it was found that the column height achievable through capillary action alone within the fiber bundle or cartridge port was insufficient to enter the region of the coil in order to achieve vaporization. Temperatures measured on the fiber bundle/coil or cartridge/coil surface are in each case above the boiling point of the materials tested (Table 2). Unlike standard vaporizers, the rate of vaporization is much lower, and the calculated coil surface temperature based on electrical power consumption is much higher in ereptiospiration.

Using the fiber bundle and coil system, ereptiospiration of coconut oil, lanolin, and acetaminophen could be visualized due to the presence of small light scattering particulates. Similarly, coconut oil produced visual evidence of ereptiospiration using the cartridge system. Ereptiospiration of the alcohols, as well as methyl ethyl ketone and water, did not generate observable aerosols and was instead observed using the cartridge system only by the presence of an audible “hissing” sound.

In order to determine the average rate of ereptiospiration, data was obtained using only the stainless steel fiber bundle/coil system. Coconut oil, lanolin, and acetaminophen, which are two solids and a viscous liquid at room temperature, were studied and the individual experimental conditions and observed rates are given in Table 3. In order to compare ereptiospiration with a standard heating method to generate vapor or aerosols, samples of all three substances were heated in a beaker or test tube in order to achieve uniform heating of the material at increasingly higher temperatures up to the temperatures where decomposition is known to take place.

During equilibrium heating of the samples, degradation was observed for all three materials: coconut oil, lanolin and acetaminophen. Coconut oil produced a dark smoke above 177 °C. Acetaminophen transforms from a powder to an oily liquid upon heating and produces a yellow smoke, leaving a dark liquid in the beaker. Lanolin showed evidence of darkening shortly after it is melted, similarly providing a dark smoke. Interestingly instability of lanolin at higher temperatures is also noticeable over long periods of ereptiospiration with the fiber bundle/coil due to the heating via thermal conduction through the coil. In fact, it was observed that lanolin near the warmest zone of the fiber bundle darkened, while the ereptiospirated material appeared to be unaffected.

**Figure 3:**
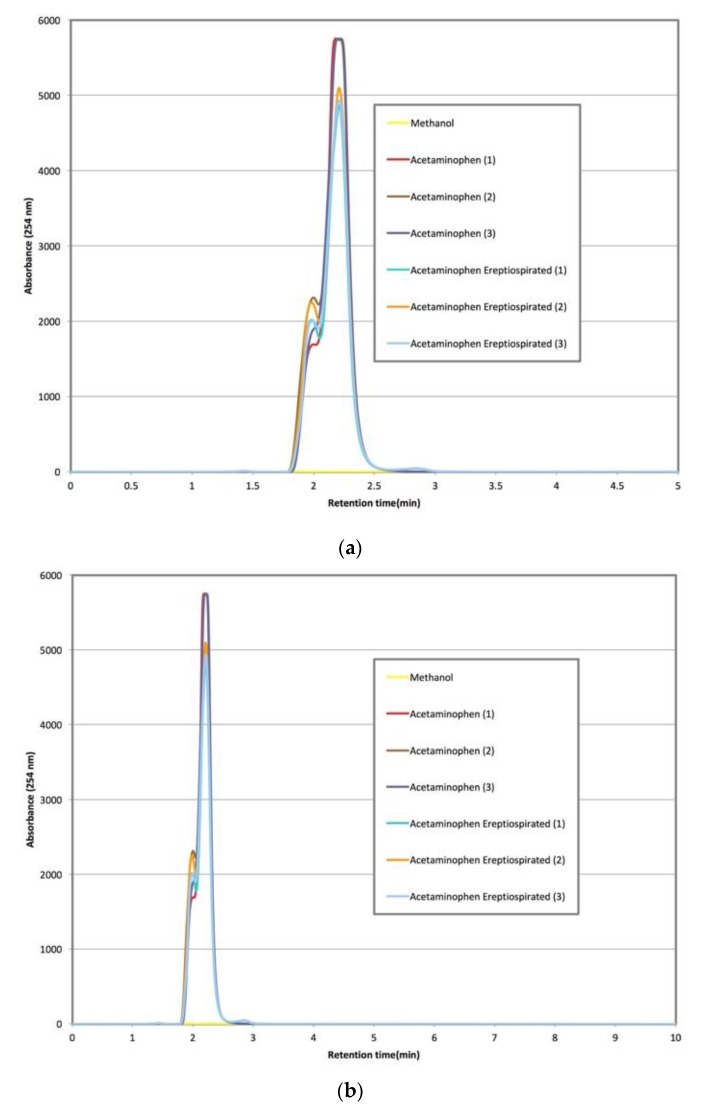
HPLC data for methanol, acetaminophen, and acetaminophen collected after ereptiospiration. Image on the top (**a**) shows the chromatogram for the time period of 0–5 min, while the image on the bottom (**b**) shows the same chromatogram for the full run time of 0–10 min. Methanol (yellow trace) does not show a peak while the ereptiospirated acetaminophen and acetaminophen from the reagent container showing peaks with the same retention time and very similar peak shape and relative heights, indicated that no chemical change was detected [16]. Note that in reference [16] using similar HPLC conditions, increased peak width change is considered to be an especially good indicator of chemical changes.

**Figure 4:**
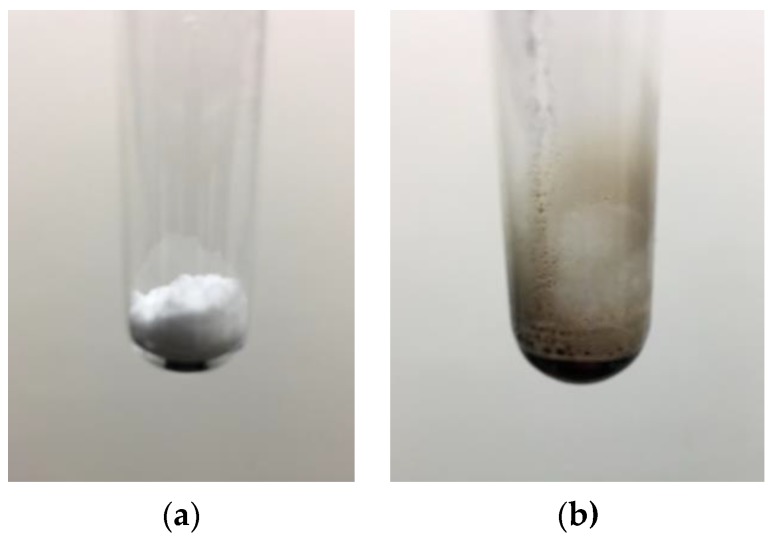
Image of acetaminophen before (**a**) and after heating with a hot air gun at 300 °C for 2 s (**b**). Clear evidence of thermal degradation is noted in panel (**b**).

During ereptiospiration testing of acetaminophen, the powdered analgesic was first lightly melted prior to application on the fiber bundle where it remained a liquid after cooling to room temperature. This is presumed to be a quasi-equilibrium state, and in fact after a few hours acetaminophen will revert to back to its large monoclinic prism form. However, for the purpose of demonstrating the ereptiospiration process, the material was used while in the liquid form since it is easier to work with and transfer quantitative. Vapor collected from these trials was analyzed using HPLC to assess whether there is a detectable level of degradation (Figure 3), which would be observed by a change in the shape of the peak and slight shift in the retention time [16]. Visual confirmation of the thermal lability of the acetaminophen powder is shown in Figure 4 where brief exposure to hot air at 300 °C led to rapid charring of the originally white powder.

For acetaminophen, vapor was collected using a cooled polymer mesh, watch glass, or stainless steel cup held over the coil. Determination of vaporization rates was performed for all three substances by weight difference with a Sartorius balance on a marble table providing repeatable measurements within 0.1 milligrams. In order to ensure that weight differences were due to vaporization, controls with no electrical power to the fiber/coil system with and without coconut oil, lanolin, or acetaminophen were also performed.

## 4. Discussion

Within seconds of powering the heating coil, we directly observed aerosol leaving the heating coil, or indirectly detected the presence of vaporized materials as they deposited onto the thin film sample collector. Unlike solvent-primed vaporization where the formation of a relatively high amount of vapor creates and the action that draws more liquid via capillary action followed by transpiration, a pressure difference in the air-filled stainless steel porous structure creates the priming action and continues to draw more material into the coil. This is primarily due to a combination of higher coil operating temperatures and the use of finer pores. The process also appears to be self-regulating in the sense that the rate depends upon flow of material through the finer pores rather than the electrical power supplied or the presence of compound in the porous cavities (see Table 3 which shows a weak correlation between power and vaporization rates). This suggests that the flow rate of compound into the vaporization zone near the heating element is dictated by Knudsen or transitional Knudsen flow rather than continuum flow as described by the Hagen–Poiseuille equation. In order to explore this concept further, calculation or estimation of the heating element surface temperature and the Knudsen number for the experimental conditions are useful (see Appendix A for more details).

Kanthal A1 wire is limited to an operating temperature of approximately 1400 °C and melts at 1500 °C. Estimation of the temperature of the 34-gauge wire based on its electrical properties and the voltage used with a measured current of 1.6–2 amperes, indicates that in air, a coil surface temperature of between 800 and 900 °C is achieved in our experiments. Moreover, based on steady-state heat transfer using a natural convection heat transfer coefficient of 1000 W/m^2^-K and the range of power used, we calculate that the wire surface temperature under most of the conditions observed is closer to 900 °C. Because the coil has such a low thermal mass, even though there is some heat conducted to the porous stainless steel structures, infrared and thermocouple measurements do not approach values much above 60 °C a short distance away from the coil. This is consistent with our calculation based on the Laplace equation for heat conduction through a stagnant fluid in that the high temperature zone is nearly entirely confined to a region of about twice the wire radius from the surface, which is about 0.075 mm. Thus, the temperature gradient is estimated to be on the order of 5000–5500 K/mm. These calculations are needed, as stated above, since a standard thermocouple probe has too much thermal mass and too large of a bulb size to properly record the true surface temperature. Instead, in the zone that corresponds to the coil surface, the maximum temperature recorded is much less than 900 °C. Moreover, our calculation of the thickness of the heating zone is conservative since air currents in the room, due to the use of a fume hood, would increase the heat transfer coefficient and thus decrease the distance of the heated zone even further.

Using the value of 900 °C and normal atmospheric pressure, the mean free path of air at the surface of the heating element is estimated to be 0.4 micrometers. The stainless steel wool, whose fibers are 35 micrometers in diameter, can create pores as small as a few micrometers in diameter, but is generally expected to have pores of an average size of 30 micrometers. For this porous structure, the Knudsen number can be in the transition region range between 0.01 and 0.1, which suggests that the pressure difference [9] may be very modest and on the order of 100–1000 pascal units. For the 3-D printed porous structure, while the pores are much fewer and much larger, we have observed that the heating element creates a pressure differential causing small amounts of alcohol liquid to be aspirated up into the chamber while slightly larger amounts of alcohol generate too much vapor and cool the coil sufficiently so that no aspiration occurs. Based on measurements of the capillary rise in the porous 3-D printed structure, we estimate that coconut oil (after warming so that it is in the liquid form) and alcohols will move up the capillary entrance into the bottom of the structure (about 8–10 mm). Similarly, capillary action for the stainless steel wool has also been measure to be about 10 mm for coconut oil as well (Table 2). However, there is no evidence of coconut oil or lanolin vaporization using an initial lower power (3 volts at 1 amp, with a resultant coil surface temperature of about 400 °C). This indicates that capillary action is insufficient to move the compound into the heating zone, and the pressure difference caused by the presence of higher temperature is needed in order to create the initial delivery of the compound to the heating zone.

An additional consideration of the ereptiospiration process is the time that the materials spend in the heated zone. An estimate of the time that compounds spend in the zone near the heating element is based on the typical rate of roughly between 1 and 40 mg/min observed, and the temperature profile determined using the Laplace equation. It is reasonable to assume that compounds will vaporize before reaching the heating element surface and may only travel for approximately 0.01–0.02 mm within the zone since the fiber bundle or 3-D printed cartridge is quite porous. Based on the very low surface area of the heating element, assuming a constant velocity, and using the principle of conservation of mass, a reasonable time estimate for the ereptiospiration processes that we generated is between 0.1 and 1 milliseconds. The strongest chemical evidence provided that an otherwise relatively labile compound can be vaporized without chemical alteration using ereptiospiration is given in the HPLC analysis of acetaminophen (Figure 3). Studies of the thermal stability for acetaminophen [14] have reported that at the temperatures reached in the heated zone, acetaminophen can degrade in less than a second, and potentially as rapidly as 0.4 milliseconds [14]. The HPLC retention times of multiple acetaminophen samples collected do not indicate new peak shape, extra peaks, or retention time shifts, indicating that the material appears to have the same chemical properties after ereptiospiration [16]. In sharp contrast to heating during ereptiospiration, in Figure 4 acetaminophen undergoes easily observable degradation within a short period of time (2 seconds) when exposed to a temperature (300 °C) which is much lower than the temperatures reached by the coil. Thermal aerosols made by batch heating [6] also indicate that short heating at high temperatures circumvents problems of degradation of labile pharmaceuticals. However, due to the batch nature of this type of rapid film heating, heating for about 500 milliseconds for a 10-milligram dose is required in order to supply the amount of medicine needed [6]. The advantages of using a flow system become apparent in that while the target dose is applied over a longer period of time, the material is subject to higher temperatures for much shorter times and the system can be operated as long as needed to achieve the therapeutic dose.

The technology of drug delivery continues to generate much research interest due to a variety of unmet needs including: accurate and consistent dosing, improving efficacy, maintenance of therapeutic levels, mitigation of side effects, alternatives to oral and parenteral routes, and faster relief, to name a few. Of particular recent interest has been in developing portable pulmonary delivery methods since the peripheral lung has a very large epithelial surface area of about 100 square meters that is less than 1 micrometer thick [17]. A growing list of nebulizers, solid particle propellant systems, surface acoustic wave devices, microfluidic platforms and new particle technologies are being marketed or under development to deliver therapeutics into the deep lung.

However, reaching the lung tissue in an effective and efficient manner is not the only step to ensuring an optimal delivery of a specific pharmaceutical. Post-particle deposition of active drug, including problems with dissolution and absorption, can affect the rate of achieving therapeutic blood concentration levels and the speed at which it is eliminated by the body [17]. Ereptiospiration may play an important role in delivering thermally-labile pharmaceuticals where pure particles can increase the speed of their action and enhance effectiveness. We envision that Central Nervous System acting pharmaceuticals used to relieve pain, such as acetaminophen, ibuprofen, and other NSAIDs, may be advantageous to deliver in this manner. From a patient needs perspective, there is a broad category of pharmaceuticals that would be desired for faster action due to acute conditions including those that alleviate anxiety or panic attacks, gastrointestinal discomfort, and allergic reactions.

Given the potential for a wide range of pharmaceuticals, molecular characteristics that would be amenable to ereptiospiration can be conjectured based on Alexza Pharmaceuticals’ [6] experience with particle formation using rapid heating of an amorphous film where from 400 pharmaceuticals tested they report that 200 have shown some vaporization feasibility. From the model compounds we have tested and from the technology described for thin film vaporization [6], we predict that the 200 compounds processed as a thin film can also be made into an amorphous solid or gel on one end of a porous cartridge or fiber bundle and heated rapidly to eject particles through ereptiospiration. At the shorter time of high heat exposure that occurs in the flow system, there may also be more pharmaceuticals amenable to delivery as a pure particle to the lungs.

## 5. Conclusions

High thermal gradients created by a thin heating coil and a porous network can generate the conditions to expel or vaporize material that is liquid or solid at room temperature into the air. Experiments based on using a stainless steel fiber bundle and a 3-D printed steel structure containing pores illustrate how the process, called ereptiospiration, combines thermal transpiration with rapid heating in order to vaporize thermally labile materials before degradation is observed to occur. Coconut oil and lanolin were found to create particles in the vapor phase that do not appear to have been combusted, as is seen when these substances are heated in a beaker or test tube. Acetaminophen was also observed to emit particles into the vapor phase that do not appear to have undergone oxidative reaction, and when analyzed using HPLC, the same retention time and peak shape as the original sample are given, with no additional peaks. This exploration of the ereptiospiration process indicates the potential for creating a continuous system of generating thermal aerosols, as compared to those formed by the batch-wise processes of ultra-fast scanning calorimetry or thin film flash vaporization. Finally, this study suggests there is potential to use thermal transpiration under high temperature gradients in order to process or deliver thermally-labile materials. We recommend that a wider study of thermally-labile materials using specially designed porous structures would be of value to identify the kinetics of thermal degradation and provide useful information on how to control heating in order to maximize the rate of vaporization, while avoiding chemical alteration of the materials being vaporized.

## Figures and Tables

**Table 1:** Properties of substances used to demonstrate ereptiospiration.

Substance	Molecular Weight (g/mol)	Density (kg/m^3^)	Surface Tension * at (20 °C) (N/m)	Viscosity (mPa-s)
Methanol	32.04	792	2.207 × 10^−2^	0.594 (27 °C)
Ethanol	46.06	789	2.197 × 10^−2^	1.095 (27 °C)
Isopropanol	60.096	803	2.3 × 10^−2^	2.1 (25 °C)
1-Butanol	74.12	810	2.42 × 10^−2^	2.53 (27 °C)
t-Butanol	74.12	780	2.07 × 10^−2^	3.35 (27 °C)
Methyl ethyl ketone	72.11	805	2.48 × 10^−2^	0.43 (27 °C)
Water	18.015	1000	7.197 × 10^−2^	1.0038 (20 °C)
Coconut oil (caprylic acid and capric acid)	144.21	924	1.748 × 10^−2^	13.6–14.5 (54 °C)
Olive oil (oleic acid)	282.468	850	1.0 × 10^−2^	20.5 (54 °C)
Eucalyptus oil (Eucalyptol)	154.25	912	7.91 × 10^−3^	33.7 (27 °C)
Lanolin	unspecified	933	unspecified	(50 °C)

* Values supplied by literature reference [15].

**Table 2:** Summary of information on substances testing with the porous network/coil systems.

Substance	Meniscus Height (mm) in 1.5-mm I.D. Capillary	Average Mass Drawn into Fiber Bundle (kg)	Average Mass Drawn into Cartridge (kg)	Column Height in Cartridge (mm)	Coil Temperature (°C, Measured on Cartridge)
Methanol	8.5	1.05 × 10^−5^	5.23 × 10^−6^	8.4	185
Ethanol	8.5	7.73 × 10^−6^	6.83 × 10^−6^	11.0	173
Isopropanol	11.5	6.16 × 10^−6^	8.50 × 10^−6^	13.5	191
1-Butanol	9.0	1.32 × 10^−5^	9.97 × 10^−6^	15.7	201.5
t-Butanol	10.5	6.53 × 10^−6^	5.40 × 10^−6^	8.81	111.8
Methyl ethyl ketone	9.0	1.16 × 10^−5^	1.34 × 10^−5^	21.2	146
Water	6.5	5.66 × 10^−6^	3.27 × 10^−6^	4.16	135
Coconut oil	8.5 (50 °C)	1.01 × 10^−5^	7.60 × 10^−6^	10.5	102
Olive oil	8.5	7.96 × 10^−6^	6.17 × 10^−6^	9.24	130
Eucalyptus oil	13.5	1.01 × 10^−5^	7.77 × 10^−6^	10.8	182.7

**Table 3:** Compilation of operating conditions and ereptiospiration rates for the fiber bundle/coil system for acetaminophen, lanolin, and coconut oil.

Substance	Applied Voltage, V	Average Current, A	Duration, min	Average Rate, mg/min
Acetaminophen	4	1.6	5	2
	4	1.7	5	0.5
	4	1.8	10	3
	4	1.6	5	2
	4	1.5	15	1
*Total Time*	-	-	*40*	*2*
*Overall Average Rate*	-	-	-	*1*
*S.D. of Rate*	-	-	-	-
Lanolin	3	1.2	48	1.6
	3	1.6	11	26
	3	1.3	10	6.1
	3	1.3	15	8.1
	3	1.4	15	16
	3	1.4	15	14
*Total Time*	*-*	*-*	*114*	*-*
*Overall Average Rate*	*-*	*-*	*-*	*12*
*S.D. of Rate*	*-*	*-*	*-*	*8*
Coconut Oil	4	1.1	22	11
	4	1.3	20	30
	4	1.3	20	15
	4	1.4	20	28
	4	1.1	20	38
	4	1	20	30
	4	1	20	44
	3.5	1.1	20	21
	3.4	0.9	20	13
	4.1	N.A.	10	43
	4.1	N.A.	10	41
	4.1	N.A.	10	43
	4.1	N.A.	10	51
	4.1	N.A.	10	44
	4.1	N.A.	10	31
	4.1	N.A.	10	47
	4	N.A.	10	23
	4	N.A.	10	31
	4	N.A.	10	47
	4	N.A.	10	23
	4	N.A.	10	31
	4	N.A.	10	43
	4	N.A.	10	46
	4	N.A.	10	43
	4	N.A.	10	15
*Total Time*	-	-	*342*	-
*Overall Average Rate*	-	-	-	*33*
*S.D. of Rate*	-	-	-	*12*

## Appendix A.

### A.1. Calculation of Coil Surface Temperature

The heating coil used for the study was a Kanthal A1 wire 34 gauge. Manufacturer specifications indicate that the average wire diameter is 0.16 mm, and this value was verified in the laboratory using a digital micrometer. Other relevant specifications provided by the manufacturer and distributors gives a maximum operating temperature of 1400 °C [18], an electrical resistivity (R) of 21 ohms/ft [19] for a 34-gauge wire (which increases at most by 3% at the maximum operating temperature), a specific heat capacity of 460 J/kg-K [18], density of 7100 kg/m^3^ [19], and a thermal conductivity (k) ranging from 11 to 35 W/m-K [19] at between 50 and 1400 °C.

Two calculations are needed to determine the temperature that the coil achieves. First, assuming natural convection with a heat transfer coefficient of 200 W/m^2^-K [20], the Biot number for the wire is estimated to be:

$$Bi=\frac{hD}{k}\approx 1.45\;\times \;10^{-3}\ll 0.1$$

Thus the entire material is at a uniform temperature. Even with the lowest value of natural convection heat transfer, the Biot number is much less than 0.1, so we can assume that a calculation of the power supplied to the coil can be related to a uniform coil temperature.

In order to determine the coil surface temperature, the power supplied is equated to the loss of energy due to the surrounding air:

$$V^{2}R=hA\left(T_{c}-T_{air}\right)$$

Using the typical power supplied to the coil is of between 5 and 5.5 watts, given a coil length of 45 mm and a heat transfer coefficient of 200 W/m^2^-K, we estimate that coil surface temperatures can range from 1100 to 1200 °C. Given the power and coil dimensions, this calculation of temperature is similar to a Nichrome wire coil temperature estimation of between 900 and 1000 °C [20].

### A.2. Estimation of Temperature Profile near the Coil

Due to the extremely low thermal mass of the coil, it is reasonable to assume that the temperature drops rapidly away from the coil surface to the surrounding air and some to the exterior of the stainless steel fiber bundle. In order to understand how the pressure difference that transports material through the fiber bundle is created by the thermal gradient, it would be useful to estimate the length over which the temperature drops from the coil surface to the measured fiber bundle temperature of between 40 and 50 °C, when measured away from the coil-fiber bundle connection.

One reasonable assumption is to ignore the heat transfer to the fiber bundle and assume that there is heat transferred by conduction to a shell of air surrounding the coil.

At steady-state, the heat supplied by the electrical power is dissipated through a shell of surrounding air assuming that no radiative heat transfer occurs:

$$\dot{Q}=\frac{2\pi k(T_{1}-T_{2})}{ln\left(\frac{r_{2}}{r_{1}}\right)}$$

The objective of this calculation is to determine the relative ratio of the outer to inner layer, which for the properties and temperatures measured yield a (*r*_2_/*r*_1_) value of about 2. Since the radius of the coil is very small, the value of *r*_2_ is about 0.15 mm. The temperature at a distance away from the center of a cylinder for steady-state conduction is:

$$T=\frac{T_{2}-T_{1}}{ln\left(\frac{r_{2}}{r_{1}}\right)}ln\left(r\right)+\frac{T_{1}ln\left(r_{2}\right)-T_{2}ln\left(r_{1}\right)}{ln\left(\frac{r_{2}}{r_{1}}\right)}$$

we can then estimate that the temperature gradient for the region where the fiber temperature reaches about 50 °C which is on the order of 5500–5800 K/mm depending upon the coil resistance and voltage supplied since those parameters vary slightly with each experiment.

### A.3. Calculation of Knudsen Number (λ/L) in order to Estimate the Pressure Difference

Normally a fiber or capillary bundle exposed to the ambient atmosphere would have a very low Knudsen number (<0.1) unless the width of the openings is on the order of a nanometer. Very low Knudsen numbers indicate that the air would behave based on a continuum relationship given by the Hagen–Pousielle equation. However, given the very large temperature gradient present due to the coil, there are networks that could have higher Knudsen numbers for micrometer-sized openings.

The mean free path of air at 1000 °C is approximately 4 × 10^−5^ m. Due to the use of extra fine stainless steel fibers compressed to form a tight bundle, openings can be on the order of 30 micrometers or as small as 5–10 micrometers. This suggests Knudsen numbers of 0.02–0.1 are possible in this system. This range suggests that the system will be in the transitional regime with a pressure difference generated of 300–5000 pascals, based on the equation by Howard [9]:

$$\frac{∆P}{P_{avg}}=\frac{3.8\;Kn^{2}}{1+4K_{n}}\frac{∆T}{T_{avg}}$$
